# Impact of subspecialty elective exposures on outcomes on the American board of internal medicine certification examination

**DOI:** 10.1186/1472-6920-12-94

**Published:** 2012-10-12

**Authors:** Victoria K Shanmugam, Katina Tsagaris, Amber Schilling, Sean McNish, Sameer Desale, Mihriye Mete, Michael Adams

**Affiliations:** 1 Department of Medicine, MedStar Georgetown University Hospital, 3800 Reservoir Road, NW Washington, DC, 20007, USA; 2 Division of Rheumatology, Immunology and Allergy, MedStar Georgetown University Hospital, 3800 Reservoir Road, NW Washington, DC, 20007, USA; 3 Division of Biostatistics and Epidemiology, MedStar Health Research Institute, 6525 Belcrest Road, Suite 700, Hyattsville, MD, 20782, USA

**Keywords:** Resident education, Gender, Elective, Subspecialty, Graduate medical education

## Abstract

**Background:**

The American Board of Internal Medicine Certification Examination (ABIM-CE) is one of several methods used to assess medical knowledge, an Accreditation Council for Graduate Medical Education (ACGME) core competency for graduating internal medicine residents. With recent changes in graduate medical education program directors and internal medicine residents are seeking evidence to guide decisions regarding residency elective choices. Prior studies have shown that formalized elective curricula improve subspecialty ABIM-CE scores. The primary aim of this study was to evaluate whether the number of subspecialty elective exposures or the specific subspecialties which residents complete electives in impact ABIM-CE scores.

**Methods:**

ABIM-CE scores, elective exposures and demographic characteristics were collected for MedStar Georgetown University Hospital internal medicine residents who were first-time takers of the ABIM-CE in 2006–2010 (n=152). Elective exposures were defined as a two-week period assigned to the respective subspecialty. ABIM-CE score was analyzed using the difference between the ABIM-CE score and the standardized passing score (delta-SPS). Subspecialty scores were analyzed using percentage of correct responses. Data was analyzed using GraphPad Prism version 5.00 for Windows.

**Results:**

Paired elective exposure and ABIM-CE scores were available in 131 residents. There was no linear correlation between ABIM-CE mean delta-SPS and the total number of electives or the number of unique elective exposures. Residents with ≤14 elective exposures had higher ABIM-CE mean delta-SPS than those with ≥15 elective exposures (143.4 compared to 129.7, p=0.051). Repeated electives in individual subspecialties were not associated with significant difference in mean ABIM-CE delta-SPS.

**Conclusions:**

This study did not demonstrate significant positive associations between individual subspecialty elective exposures and ABIM-CE mean delta-SPS score. Residents with ≤14 elective exposures had higher ABIM-CE mean delta-SPS than those with ≥15 elective exposures suggesting there may be an “ideal” number of elective exposures that supports improved ABIM-CE performance. Repeated elective exposures in an individual specialty did not correlate with overall or subspecialty ABIM-CE performance.

## Background

Medical knowledge is an Accreditation Council for Graduate Medical Education (ACGME) core competency. Scores on the American Board of Internal Medicine Certification Examination (ABIM-CE) are one of several methods used to assess medical knowledge in graduating medical residents. ACGME requirements have dramatically changed residency education over the last five years and limitations on resident duty hours implemented in 2003 have placed a burden on program directors to ensure that residents’ time is well distributed between service duties and educational experiences [1]. Concomitantly with the duty hours limitations, there has been a shift towards measurable-educational outcomes in resident training. Program directors have to balance the resident educational experience, to ensure that residents gain the required medical knowledge to pass the ABIM-CE, while also permitting resident autonomy to redirect their training experience to achieve their intended career goals [2-4].

To assist programs in evaluating resident performance, the ACGME has defined a toolbox of methods for assessing the six required competencies, including medical knowledge. Many programs have now adopted these methods which include the Internal Medicine In-Training Examination (IM-ITE), the American Board of Internal Medicine Certification Examination (ABIM-CE), standardized patients, objective-structured clinical examinations (OSCE), 360 multi-source evaluations, program director medical knowledge score, patient surveys, portfolios, oral exams and checklists [5]. Performance on the IM-ITE is a predictor of performance on the ABIM-CE [6,7] and studies have shown outcomes on the IM-ITE can be improved with conference attendance and self directed reading of electronic knowledge resources [8,9]. While standardized test performance does not encompass all of the competencies required from a graduating resident, satisfactory performance on the ABIM-CE is a goal common to all graduating residents and to all internal medicine residency programs. Therefore, it is important to assess the factors that impact resident performance on the ABIM-CE.

The choice of elective exposures is one of the few components of residency training over which individual residents maintain autonomy, yet there is a paucity of data to guide residents in selecting subspecialty electives. For a variety of reasons, residents may pursue several electives in one particular subspecialty but not rotate through other subspecialties at all. Prior studies have shown that subspecialty ABIM-CE performance can be improved by developing structured curricula within the elective experience [10]. However, data comparing the impact of subspecialty elective exposures and ABIM-CE performance is lacking and would be of great value to program directors as they reorganize and develop graduate medical education programs.

The primary aim of the current study is to evaluate the impact of individual subspecialty elective exposures on resident ABIM-CE scores within a university based residency program. While we predict exposure to individual subspecialty electives might be associated with subspecialty ABIM-CE performance, we hypothesize that repeated exposures in a single specialty may not further improve ABIM-CE performance.

## Methods

This study was approved by the MedStar Georgetown University Hospital (MGUH) Institutional Review Board. ABIM-CE score reports, elective exposures, and demographic characteristics were collected for all internal medicine categorical residents enrolled in the MGUH Internal Medicine residency program who took the ABIM-CE for the first time between 2006 and 2010. ABIM-CE scores were released by the program director and de-identified by an investigator not involved in medical education to ensure confidentiality. Residents who declined to release their ABIM-CE score report to the program director as well as those who had transferred into the program were excluded from the analysis due to incomplete elective exposure and ABIM-CE data.

### Demographic information

Demographic information was obtained for each resident, including gender, age at time of ABIM-CE, and whether the resident graduated from a US or international medical school.

### ABIM-CE score abstraction and calculation of delta-SPS

ABIM-CE scores include a standardized test score and a report of the total number of items correct, with a breakdown of correct responses by subspecialty. The standardized test score incorporates a weighting based on test characteristics, it is therefore not directly comparable from year to year. To accommodate for this, and to allow more accurate measure of spread of scores, the difference between the individual resident standardized score and the standardized passing score for the exam year was calculated (the delta-standardized passing score or delta-SPS). Raw scores were additionally used to compute percentage of items correct in each subspecialty.

### Elective exposure data collection

Using on-call schedule records, elective exposure data was collected on all residents included in the study. To allow consistent comparisons between electives of differing duration, an elective exposure was defined as a two-week period in the respective subspecialty. Thus, a resident completing a four-week elective in gastroenterology would be considered to have had two two-week exposures to gastroenterology. Residents who transferred into the MGUH residency program did not have accurate data on elective exposures prior to enrollment in the MGUH program; therefore these residents were excluded from further analysis.

### Program director medical knowledge score

The ABIM requires internal medicine program directors to submit ratings of their residents in overall clinical competence and its essential components prior to the participation in the ABIM-CE. These evaluations are made on a 9-point Likert scale with 1–3 considered “unsatisfactory,” 4–6 considered “satisfactory,” and 7–9 considered “superior.” Candidates with program director medical knowledge Scores of less than 4 are not eligible to participate in the examination. The MGUH program director medical knowledge scores were available for residents sitting the ABIM-CE in years 2007–2010 and were compared to corresponding resident ABIM-CE scores.

### Statistical analysis

Descriptive statistics were calculated for sociodemographic characteristics. D’Agostino and Pearson omnibus normality testing was performed to assess data distribution, and associations were analyzed using unpaired t-tests, one-way analysis of variance (ANOVA), chi-squared test and linear regression using GraphPad Prism version 5.00 for Windows (GraphPad Software, San Diego, CA, USA). *P*-values less than 0.05 were considered significant. In the five years under study 7 residents failed the ABIM-CE (5 women, 2 men) giving a pass rate of 95%, with 90% pass rate for women residents and 97% pass rate for men (p=0.07 chi-square test).

## Results

Of the 152 residents enrolled in the MGUH internal medicine residency who were first-time takers of the ABIM-CE from 2006–2010, 131 had paired elective exposure and ABIM-CE score data available.

### Sociodemographic characteristics

Sociodemographic characteristics were comparable for each annual resident cohort (Table 1). There was no significant association between ABIM-CE score and age. While the annual cohorts had similar age distributions, the male to female ratio was higher in 2006–2008 but more evenly distributed in 2009–2010. For the whole group, the mean delta-SPS was lower for women than men (mean 117.4 compared to 146.6, p=0.0485, Table 2).

**Table 1:** Demographic variables for each annual cohort from 2006 to 2010

**Year**	**2006**	**2007**	**2008**	**2009**	**2010**
n	25	27	21	31	27
Mean age, years (range)	30 (26–35)	31 (29–34)	31 (28–38)	31 (28–39)	31 (29–35)
Number of men	19	17	13	15	12
Number of women	6	10	8	16	15
Male: Female ratio	3.17	1.7	1.63	0.94	0.80
Number of International Medical Graduates (IMG)	1	2	2	2	2
Number of residents passing ABIM-CE (%)	25 (100%)	27 (100%)	20 (95%)	28 (90%)	24 (88.9%)
ABIM-CE mean delta-SPS (mean, range)	162 (10–340)	171 (29–296)	135 (−62-339)	113 (−13-273)	96 (−28-277)
ABIM-CE standardized passing score	351	351	370	370	370
Number of residents sitting ABIM-CE ≥1 year after peer group	2	3	0	2	0
Mean number of elective exposures per resident (range)	14 (8–18)	15 (8–20)	16 (12–25)	17 (8–24)	19 (13–25)

Pass rate for IMG was 100% compared to 94% for US graduates (p=1.0, Fisher’s exact test).

**Table 2:** **Mean** (**95**% **CI**) **delta**-**SPS by demographic characteristics**

	**n**	**Mean delta**-**SPS**	**95%CI**	***p value***
Female	55	117.4	96.2-138.5	***0***.***0485***
Male	76	146.6	126.9-166.2	
Age ≥31	67	133.5	111.8-155.3	*0.91*
Age ≤30	64	135.1	115.6-154.6	
Number of elective exposures ≤14	44	143.4	122.5-164.3	*0.051*
Number of elective exposures ≥15	87	129.7	110.4-148.9	
Number of unique electives ≤8	101	134.3	117.7-150.9	*1.00*
Number of unique electives ≥9	30	134.2	102.9-165.4	

*P*-*values* calculated using two-tailed *t*-test. Male:Female ratio for the entire cohort was 1.38. Male:Female ratio for the group with ≤14 elective exposures was 1.32 compared to 1.41 for the group with ≥15 elective exposures (p=ns).

### International medical graduates

The number of international medical graduates (IMG) in the MGUH program was small (n=9), but it was similar in all years studied. IMGs did not exhibit significant differences in mean delta-SPS or ABIM-CE pass rate compared to US medical graduates in this program.

### Comparison of ABIM-CE score and number of elective exposures

The mean number of elective exposures per resident has been steadily increasing from 14 in 2006 to 19 in 2010. There was no linear correlation between total number of elective exposures, or number of unique elective exposures and ABIM-CE mean delta-SPS (Table 1). Residents with fewer than 14 elective exposures had higher ABIM-CE mean delta-SPS than those with 15 or more elective exposures (mean delta-SPS 143.4 compared to 129.7, p=0.051) suggesting there may be an “ideal” number of elective exposures that supports improved ABIM-CE performance, but above which performance does not further improve.

### Comparison of ABIM-CE score and individual subspecialty elective exposures

ABIM-CE mean delta-SPS (95% CI) for residents exposed compared to unexposed to particular subspecialty electives is shown in Table 3, broken down by annual cohort, and for the entire dataset. There was a negative association between cardiology elective exposure and mean delta-SPS (mean 128.6 for exposed residents, compared to mean 170.1 for unexposured, p=0.05). In the 2008 cohort both pulmonary and rheumatology electives were associated with significantly higher mean delta-SPS. When data from all cohorts was combined, no significant difference was seen in ABIM-CE mean delta-SPS for exposed compared to unexposed residents for any of the individual subspecialty electives.

**Table 3:** **ABIM**-**CE performance by year group and elective exposure**

		**2006 (n=25)**		**2007 (n=27)**		**2008 (n=21)**		**2009 (n=31)**		**2010 (n=27)**		**TOTAL**	
		**n**	**Mean delta-SPS (95% CI)**	**n**	**Mean delta-SPS (95% CI)**	**n**	**Mean delta-SPS (95% CI)**	**n**	**Mean delta-SPS (95% CI)**	**n**	**Mean delta-SPS (95% CI)**	**n**	**Mean delta-SPS (95% CI)**
**Cardiology**	Exposed	19	158.9	21	172.3	17	122.5	31	112.7	25	92.6	113	128.6
			(123.4 - 194.5)		(137.4-207.3)		(80.0-165.1)		(82.7-142.8)		(56.4-128.9)		(112.7-144.5)
	Unexposed	6	172.8	6	165.5	4	188.3	0	-	2	139.5	18	170.1
			(111.5-234.2)		(90.2-240.8)		(39.4-337.1)				(69.6-209.4)		(137.6-202.6)
	*p*		*0*.*68*		*0*.*85*		*0*.*18*		-		*0*.*47*		***0***.***05***
**Endocrinology**	Exposed	17	149.0	15	192.7	14	141.1	19	104.8	23	104.7	88	134.1
			(108.7-189.3)		(145.8-239.5)		(81.8-200.5)		(64.6-145.0)		(67.9-141.6)		(114.9-153.3)
	Unexposed	8	190.5	12	143.5	7	122.9	12	125.3	4	46.5	43	134.8
			(161.8-219.2)		(111.7-175.3)		(83.7-162.0)		(73.5-177.0)		(−78.1-171.0)		(113.7-155.8)
	*p*		*0*.*17*		*0*.*09*		*0*.*66*		*0*.*51*		*0*.*21*		*0*.*96*
**Gastroenterology**	Exposed	9	143.7	16	168.6	12	152.2	22	109.3	23	105.6	82	129.9
			(95.5-191.9)		(130.4-206.8)		(85.4-218.9)		(75.3-143.4)		(67.7-143.4)		(111.3-148.4)
	Unexposed	16	172.8	11	174.1	9	112.2	9	121.0	4	41.8	49	141.7
			(133.9-211.6)		(119.3-228.9)		(73.7-150.7)		(45.8-196.2)		(−33.7-117.2)		(118.0-165.5)
	*p*		*0*.*33*		*0*.*85*		*0*.*31*		*0*.*73*		*0*.*17*		*0*.*43*
**Hematology**	Exposed	4	136.3	14	167.4	12	139.3	15	115.5	20	111.6	65	131.1
			(44.9-227.6)		(122.3-212.5)		(93.1-185.4)		(71.6-159.5)		(70.7-152.4)		(111.3-151.0)
	Unexposed	21	167.2	13	174.5	9	129.4	16	110.1	7	52.0	66	137.4
			(134.6-199.9)		(130.2-218.7)		(47.4-211.5)		(63.9-156.3)		(−8.9-112.9)		(115.9-158.9)
	*p*		*0*.*43*		*0*.*81*		*0*.*8*		*0*.*86*		*0*.*11*		*0*.*67*
**Infectious Diseases**	Exposed	24	162.2	24	167.0	16	135.7	24	111.5	23	94.9	111	134.5
			(132.2-192.2)		(135.5-198.5)		(101.0-170.4)		(76.0-147.0)		(61.3-128.5)		(119.6-149.5)
	Unexposed	1	164.0	3	201.0	5	133.0	7	116.9	4	103.3	20	133.2
					(2.1-399.9)		(−49.0-315.0)		(142.4-191.3)		(−114.7-321.2)		(83.5-182.8)
	*p*		-		*0*.*47*		*0*.*95*		*0*.*88*		*0*.*36*		*0*.*95*
**Oncology**	Exposed	8	150.9	13	167.4	12	139.3	15	115.5	20	111.6	69	133.1
			(92.0-209.7)		(122.3-212.5)		(93.1-185.4)		(71.6-159.5)		(70.7-152.4)		(113.8-152.4)
	Unexposed	17	167.6	14	174.5	9	129.4	16	110.1	7	52.0	62	135.6
			(131.5-204.0)		(130.2-218.7)		(47.4-211.5)		(63.9-156.3)		(−8.9-112.9)		(113.3-157.9)
	*p*		*0*.*58*		*0*.*81*		*0*.*80*		*0*.*86*		*0*.*11*		*0*.*87*
**Nephrology**	Exposed	17	161.9	16	172.4	14	169.6	17	119.2	13	62.9	77	129.9
			(135.1-188.6)		(134.4-210.5)		(101.4-237.7)		(76.2-162.2)		(22.3-103.5)		(111.5-148.3)
	Unexposed	8	163.1	11	168.5	7	117.8	14	104.9	14	126.9	54	140.6
			(77.8-248.4)		(113.3-223.6)		(66.0-169.6)		(57.4-152.3)		(74.4-179.5)		(116.6-164.5)
	*p*		*0*.*97*		*0*.*89*		*0*.*20*		*0*.*63*		***0***.***049***		*0*.*48*
**Pulmonary**	Exposed	19	164.4	19	181.5	15	158.3	21	117.1	19	99.8	93	143.0
			(127.1-201.8)		(144.4-218.5)		(113.5-203.2)		(53.8-145.8)		(125.1-160.9)		(76.5-81.1)
	Unexposed	6	155.5	8	145.5	6	76.8	10	103.5	8	87.4	38	112.9
			(114.1-196.9)		(90.5-200.5)		(−3.6-157.3)		(37.7-137.1)		(89.4-136.5)		(69.8-76.7)
	*p*		*0*.*79*		*0*.*26*		***0***.***048***		*0*.*67*		*0*.*74*		*0*.*06*
**Rheumatology**	Exposed	18	150.0	19	176.9	12	171.9	22	126.3	18	94.1	89	141.5
			(120.8-179.2)		(138.3-215.6)		(119.3-224.6)		(92.8-159.8)		(52.1-136)		(124.5-158.5)
	Unexposed	7	193.9	8	156.3	9	85.9	9	79.4	9	100.2	42	119.0
			(111.3-276.4)		(105.7-206.8)		(34.3-137.5)		(8.8-150.1)		(29.3-171.1)		(91.3-146.6)
	*p*		*0*.*16*		*0*.*52*		***0***.***0197***		*0*.*15*		*0*.*86*		*0*.*15*
**Primary Care**	Exposed	6	212.3	7	168.7	5	146.4	5	108.2	7	99.7	30	144.3
			(137.2-287.5)		(103.2-234.2)		(18.4-274.4)		(25.9-190.5)		(19.8-151.9)		(112.6-176.0)
	Unexposed	19	146.5	20	171.6	16	131.5	26	113.6	20	85.9	101	131.3
			(116.3-176.7)		(135.3-207.8)		(86.6-176.4)		(79.0-148.2)		(57.0-142.4)		(114.9-147.8)
	*p*		**0**.**04**		0.93		0.74		0.89		0.72		0.50

Scores are reported as difference in standardized score compared to standardized passing score (delta-SPS) reported as mean (95% confidence interval). p-values represent comparison of exposed to unexposed residents for each elective exposure analyzed using unpaired two tailed *t*-test.

### Comparison of ABIM-CE score and repeated elective exposures

ANOVA analysis comparing ABIM-CE scores in subjects with 0, 1–2 and more than 2 elective exposures in each subspecialty did not show a significant association between repeated elective exposures and improved performance on the ABIM-CE based either on the total percentage correct or the subspecialty percentage correct (Tables 4 and 5).

**Table 4:** **Mean delta**-**SPS** (**95**% **CI**) **by number of elective exposures in each specialty**

**Exposure**		**0**	**1-2**	**≥3**	***p***
Cardiology	n	18	39	74	*0*.*09*
	Mean	170.1	138.6	123.4	
	(95% CI)	(137.6-202.6)	(114.2-162.9)	(102.5-144.2)	
Endocrinology	n	43	74	14	*0*.*84*
	Mean	134.8	131.7	146.4	
	(95% CI)	(113.7-155.8)	(110.5-153.0)	(96.9-195.9)	
Gastroenterology	n	49	63	19	*0*.*64*
	Mean	141.7	132.5	121.2	
	(95% CI)	(118.0-165.5)	(114.0-151.0)	(65.6-176.7)	
Hematology	n	66	63	2	*0*.*64*
	Mean	137.4	132.7	81.50	
	(95% CI)	(115.9-158.9)	(112.8-152.6)	(NA)	
Infectious Diseases	n	20	64	47	*0*.*45*
	Mean	133.2	125.8	146.3	
	(95% CI)	(83.5-182.8)	(105.7-146.0)	(123.6-169.0)	
Oncology	n	62	68	1	*NA*
	Mean	135.6	132.5	176.0	
	(95% CI)	(113.3-157.9)	(113.0-152.0)	(NA)	
Nephrology	n	54	71	6	*0*.*06*
	Mean	140.6	136.2	55.33	
	(95% CI)	(116.6-164.5)	(117.7-154.7)	(−33.2-143.9)	
Pulmonary	n	38	79	14	*0*.*14*
	Mean	112.9	145.6	128.7	
	(95% CI)	(89.4-136.5)	(125.8-165.3)	(81.4-176.0)	
Rheumatology	n	42	82	7	*0*.*32*
	Mean	119.0	142.7	127.6	
	(95% CI)	(91.3-146.6)	(124.6-160.9)	(73.7-181.4)	
Primary Care	n	101	26	4	*0*.*28*
	Mean	131.3	152.8	89.0	
	(95% CI)	(114.9-147.8)	(118.0-187.6)	(−0.5-178.5)	

*P*-*value* calculated using one-way ANOVA.

**Table 5:** **Mean** (**95**% **CI**) **subspecialty**% **of questions answered correctly, broken down by number of elective exposures in subspecialty of interest**

**Exposure**		**0**	**1**-**2**	≥**3**	***p***
Cardiology	n	18	39	74	
	Mean (%)	79.6	78.5	81.0	*0*.*41*
	(95% CI)	(74.9-84.2)	(75.5-81.6)	(78.8-83.3)	
Endocrinology	n	43	74	14	
	Mean (%)	77.7	78.3	83.6	*0*.*19*
	(95% CI)	(74.6-80.8)	(75.5-81.1)	(80.5-86.8)	
Gastroenterology	n	49	63	19	
	Mean (%)	76.9	78.4	82.1	*0*.*17*
	(95% CI)	(73.8-80.0)	(76.1-80.8)	(77.2-86.9)	
Hematology	n	66	63	2	
	Mean (%)	81.4	80.7	91.5	*0*.*49*
	(95% CI)	(78.2-84.5)	(77.4-83.9)	(NA)	
Infectious Diseases	n	20	64	47	
	Mean (%)	80.9	79.3	81.8	*0*.*45*
	(95% CI)	(75.7-86.2)	(76.8-81.8)	(78.8-84.7)	
Oncology	n	62	68	1	
	Mean (%)	72.6	75.9	86.0	*NA*
	(95% CI)	(69.4-75.7)	(72.9-78.8)	(NA)	
Nephrology	n	54	71	6	
	Mean (%)	83.3	84.3	75.8	*0*.*27*
	(95% CI)	(76.9-86.7)	(81.6-86.9)	(54.9-96.8)	
Pulmonary	n	38	79	14	
	Mean (%)	73.2	78.6	79.7	***0***.***03***
	(95% CI)	(69.8-76.7)	(76.1-81.1)	(73.9-85.5)	
Rheumatology	n	42	82	7	
	Mean (%)	80.5	80.2	86	*0*.*35*
	(95% CI)	(77.6-83.4)	(77.8-82.6)	(80.3-91.7)	
Primary Care	n	101	26	4	
	Mean (%)	75.3	78.0	74.5	*0*.*35*
	(95% CI)	(73.7-76.9)	(74.3-81.6)	(56.8-92.2)	

p-values calculated using 1-way ANOVA.

### Comparison of elective exposure and ABIM-CE subspecialty score

Residents completing the pulmonary elective exhibited higher mean scores on pulmonary questions on the ABIM-CE (mean 78.8% for exposed residents compared to 73.2% for unexposed, p=0.0092, Table 6). No significant associations were seen between other elective exposures and subspecialty percentage correct.

**Table 6:** **Mean** (**95**% **CI**)% **correct in respective subspecialties for each elective exposure**

**Exposure**		**Not exposed**	**Exposed**	***p***
Cardiology	n	18	113	
	Mean	79.6	80.2	*0*.*81*
	(95% CI)	(74.9-84.2)	(78.3-81.9)	
Endocrinology	n	43	88	
	Mean	77.7	79.2	*0*.*48*
	(95% CI)	(74.6-80.8)	(76.7-81.6)	
Gastroenterology	n	49	82	
	Mean	76.9	79.3	*0*.*20*
	(95% CI)	(73.9-80.0)	(77.2-81.4)	
Hematology	n	66	65	
	Mean	81.4	81.0	*0*.*87*
	(95% CI)	(78.2-84.5)	(77.8-84.2)	
Infectious Diseases	n	20	111	
	Mean	80.9	80.4	*0*.*81*
	(95% CI)	(75.7-86.2)	(78.5-82.3)	
Oncology	n	62	69	
	Mean	72.6	76.0	*0*.*11*
	(95% CI)	(69.4-75.7)	(73.1-78.9)	
Nephrology	n	54	74	
	Mean	83.3	83.6	*0*.*87*
	(95% CI)	(79.9-86.7)	(80.9-86.4)	
Pulmonary	n	38	93	
	Mean	73.2	78.8	*0*.*0092*
	(95% CI)	(69.8-76.7)	(76.5-81.1)	
Rheumatology	n	42	89	
	Mean	80.48	80.64	*0*.*93*
	(95% CI)	(77.6-83.4)	(78.4-82.9)	
Primary Care	n	101	30	
	Mean	75.3	77.5	*0*.*21*
	(95% CI)	(73.6-76.9)	(74.1-80.9)	

P-value calculated using two-tailed unpaired *t*-testP-value calculated using two-tailed unpaired *t*-test.

### Program director medical knowledge score

The program director medical knowledge score was significantly associated with ABIM-CE mean delta-SPS, *r*^2^ 0.365, *p*<0.001. Despite the association between the program director medical knowledge score and ABIM-CE score, there was no gender discrepancy in the program director medical knowledge score (mean for female residents 7.06, CI 6.65-7.48, mean for male residents 7.20, CI 6.86-7.55, *p*=0.77).

## Discussion

Resident elective selection is dependent on numerous factors including career preference, subspecialty interest, real or perceived quality of subspecialty education, opportunity to secure faculty recommendation letters, desire to improve knowledge in the subspecialty, auditioning for fellowship opportunities, guidance of an advisor or program director, and even such things as ease of the schedule or rigorousness of the rotation.

Although analysis of the data from 2008 suggested a positive association between exposure to the pulmonary and rheumatology electives and improved ABIM-CE scores, analysis of subspecialty elective exposures for all years combined failed to demonstrate a statistically significant positive relationship between specific elective exposures and ABIM-CE scores. It is possible that the sample size in this study was too small to demonstrate differences in outcomes simply because the residency program studied has relatively high numbers of elective exposures and high overall pass rates on the ABIM-CE. Another factor which may have contributed to lack of association between elective exposure and ABIM-CE performance in this study is that while all major electives in our program have a formal curriculum, there is wide variation in the structure, goals and objectives of the individual electives. Some electives include both inpatient and outpatient experiences, while others focus on only one or the other. The elective experience may vary dependent on the teaching experience of the attending physician. Finally, electives vary as to inclusion of pre- and post-testing, required reading lists, and subspecialty conference exposures. Prior studies have shown that formalized elective curricula [11,12] or inclusion of elective specific multiple-choice testing program [13] improves resident performance on standardized tests of medical knowledge.

In our study participation in the cardiology elective was associated with worse performance on the ABIM-CE. This finding may reflect the nature of this elective as a more service driven elective, with a focus on repetitive, protocol driven, hospital-based experience and fewer one-on-one resident-attending interactions. Investigation of the characteristics of specific electives, such as the inpatient and outpatient focus, inclusion of reading lists, subspecialty conferences and pre- and post-testing these relationships was outside the scope of this study, but merits further investigation in a larger prospective study.

Our study did not demonstrate a statistically significant relationship between repeated elective exposures in a single specialty and ABIM-CE performance, suggesting that repeated exposure to a particular subspecialty may not offer additional improvement in medical knowledge. Although the population size limited the statistical significance of this finding, the data suggests that there may be a “threshold” number of elective exposures associated with improved ABIM-CE scores, but above which further elective experiences do not further improve ABIM-CE performance. This observation is helpful to program directors planning schedules and supports recommendations to move residency education from a structure and process based “time dependent” system towards a more competency based program where the major outcome is knowledge acquisition driven by the learner and assessed using multiple outcome measures [14].

While there is an increasing push towards self-directed learning and autonomy in selection of electives it is well recognized that residents themselves are not always good judges of their own medical knowledge [15]. As in prior studies [16], our data showed strong correlation between the program director medical knowledge score and the ABIM-CE score, reinforcing the continued predictive value of program director evaluations, and supporting use of these evaluations in guiding residents throughout residency.

An unexpected finding of this study was the statistically significant negative association between female gender and ABIM-CE performance in this program. In the United States the number of women applying to medical school is increasing [17]. Women now make up more than 50% of matriculating medical students, and 25% of practicing physicians [18]. A similar negative association between female gender and ABIM-CE performance was reported in a larger study evaluating internal medicine residents’ performance on the IM-ITE and correlating this with quality of life, burnout and educational debt [19]. Our findings suggest that female residents may experience barriers to education during residency that impact ABIM-CE performance, an observation that merits further investigation.

Our study has some limitations that warrant further discussion. The study was conducted in a single, university-based residency program with an ABIM-CE mean pass rate above the national average. It may therefore have been underpowered to show a difference in ABIM-CE score with subspecialty elective exposure. Residents in our program are permitted numerous subspecialty electives throughout the three years of training, and this may have contributed to the absence of detectable difference for high enrollment electives (such as infectious diseases). Furthermore, the observed results may not be widely applicable to training programs with fewer subspecialty elective opportunities.

It should also be noted that we studied only subspecialty exposures and did not assess reasons for elective selection or avoidance. There may be a selection bias in that residents who were interested in a subspecialty may be more likely to study in that subspecialty. Additionally, in our program, residents with poor subspecialty performance on the annual IM-ITE are counseled to participate in an elective in that subspecialty. Family and economic issues may further confound the relationship between ABIM-CE performance and elective exposures. Residents who become parents during residency may time their elective exposures to dovetail with their parental leave, and sleep deprivation and other stresses may thus impact their elective experience. Educational debt may be another confounder in the relationship with ABIM-CE performance. Prior studies have shown that educational debt is associated with lower mean IM-ITE scores [19]. In many internal medicine residencies, the somewhat lighter call schedule during electives affords residents an opportunity to take on additional “moonlighting” shifts. During the time period of this study our institution had a moonlighting policy in place, which permitted residents with high satisfactory evaluations to moonlight while on elective. This may have resulted in residents with higher educational debt taking more electives, or selecting electives with lighter duties possibly skewing the associations between elective exposures and ABIM-CE performance.

The data from Table 1 show that mean delta-SPS scores declined over the five years under study, while the number of electives taken increased. In addition to the confounders discussed above, ACGME limitations on duty hours may play a role in these observations. The ACGME changed duty hour requirements in July 2003 and July 2011, so all cohorts in the current study fell under the auspices of the 2003 requirements including: a) 80-hour limits on the resident work week; b) 30-hour limit on overnight/continuous duty shifts; c) one day in seven (averaged) free of all duties; d) no more frequent than every third night overnight call, and e) “adequate” rest periods. Since data is not available for residents taking the ABIM-CE prior to 2005 it is not possible to compare the cohort under study with a historical cohort prior to the implementation of the 2003 ACGME duty hour requirements. A further change in ACGME duty hours was implemented in 2011, so it would be interesting to see if this trend towards declining ABIM-CE performance persists in future cohorts.

We did make an attempt to investigate the effect of timing of subspecialty elective exposure on IM-ITE scores in order to compare scores before and after an elective as well as to help measure knowledge retention throughout residency. However, although residents in our program are expected to participate in this annual exam; some residents were unable to complete the exam in each of the three years of residency because of personal issues or scheduling conflicts. Due to the paucity of data we could not evaluate impact of elective exposure over time in our cohort, but this would be important to study in a larger or prospective cohort.

Residency education is continuously evolving and adapting to the learning environment. With the advent of the Next Accreditation System (NAS) [4] there will be an emphasis on the responsibility of the sponsoring institution to ensure quality of the learning environment. With this in mind it is important for residency programs to evalute the impact of elective exposures on outcomes and to identify innovations that improve the quality of these exposures.

## Conclusions

In this small retrospective study of a single university-based internal medicine residency, we did not find positive associations between subspecialty elective participation and ABIM-CE performance. Residents with fewer than 14 elective exposures had higher ABIM-CE mean delta-SPS than those with 15 or more elective exposures. Repeated subspecialty elective exposures did not correlate with either total or subspecialty score on the ABIM-CE suggesting that residents should be cautioned against repeated electives in a single specialty since it is unlikely to further broaden medical knowledge. This data should be of interest to residents planning their elective selections, and to program directors as they adapt resident education to respond to the Next Accreditation System.

## Competing interests

All authors have completed and submitted the ICMJE Form for Disclosure of Potential Conflicts of Interest. Dr. Adams is currently the Program Director of the MedStar Georgetown University Hospital internal medicine residency program.

## Authors’ contributions

VKS had full access to all the data in the study and takes responsibility for the integrity of the data and the accuracy of the data analysis. Study concept and design: VKS, KT, MA. Acquisition of data: VKS, KT, SM, AS. Statistical analysis: VKS, MM, SD. Analysis and interpretation of data: VKS, KT, MM, SD. Drafting of the manuscript: VKS, KT. Critical revision of the manuscript for important intellectual content: VKS, KT, MA, MM, SD, SM. Administrative, technical or material support: VKS, MA, SM, AS Study supervision: VKS, MA. All authors read and approved the final manuscript.

## Pre-publication history

The pre-publication history for this paper can be accessed here:

http://www.biomedcentral.com/1472-6920/12/94/prepub
